# Plasmids in environmental microbes: An annotated selection of World Wide Web sites relevant to the topics in environmental microbiology

**DOI:** 10.1111/1758-2229.13218

**Published:** 2023-11-24

**Authors:** Lawrence P. Wackett

**Affiliations:** ^1^ McKnight Professor, Department of Biochemistry, Molecular Biology & Biophysics, BioTechnology Institute University of Minnesota St. Paul MN 55108 USA

## Plasmids vary in different environments

https://journals.asm.org/doi/epub/10.1128/mbio.03191-22

Most studies of plasmids focus on traits like antibiotic resistance, which account for ~1% of plasmid genes. This study looked broadly at plasmids to better understand their composition and distribution as a function of environment.

## Plasmid transfer in communities

https://www.sciencedirect.com/science/article/pii/S1369527422000364

The studies described here investigate how microbial communities alter conjugative plasmid transfer and persistence.

## Plasmids from and for *Pseudomonas*

https://www.addgene.org/search/catalog/plasmids/?q=pseudomonas

This commercial site contains a large list of plasmids suitable for research with *Pseudomonas*. It includes plasmid maps and other useful information.

## Plasmids modulate their hosts

https://royalsocietypublishing.org/doi/10.1098/rstb.2020.0461

This opinion piece discusses the role of plasmids in vertical and horizontal transfer and how plasmids might modulate their hosts for their own benefit in some instances.

## Positive and negative selection for plasmids

https://blog.addgene.org/plasmids-101-positive-and-negative-selection-for-plasmid-cloning

The blog post here discusses the basics of using plasmids for negative and positive selection purposes.

## Plasmid stability without selection

https://www.nature.com/articles/s41467-019-10600-7

This paper describes how plasmids might persist over evolutionary time scales without having selectable markers associated with the plasmid.

## Plasmids for non‐selection use

https://pubs.acs.org/doi/10.1021/acssynbio.0c00466

This report documents that *Escherichia coli* Nissle 1917 natively contains two plasmids, the plasmids are maintained without selection, and they can be engineered for molecular engineering.

## Plasmids to deliver genes to microbiomes

https://phys.org/news/2022-12-reassemble-plasmid-genes-diverse-environmental.html

This news article describes research in which an environmentally‐derived plasmid was engineered to be highly promiscuous. It could transfer between an *Escherichia coli, Pseudomonas putida, Bacillus subtilis* and *Saccharomyces cerevisiae*.

## Mining environmental plasmids for parts

https://journals.asm.org/doi/epub/10.1128/microbiolspec.plas-0033-2014

Here is reviewed plasmids and molecular parts of vectors than can be used for engineering purposes.

## Vector database

https://www.addgene.org/vector-database/query/

The Vector database currently has 4,399 entries with information on vector backbones. It is compiled by Addgene but the resources are from third party sources and are not their commercial plasmids. There is a link to search Addgene plasmids for those interested.

## Plasmids and vector resources

http://www.geneinfinity.org/sp/sp_plasmidsandvectors.html

This page provides links to collections of plasmid and vector resources.

## Plasmid files

https://www.snapgene.com/plasmids

This site contains plasmid annotations and sequences with a focus on cloning vectors from different commercial sources.

## Plasmid detection in genomic data

https://www.ncbi.nlm.nih.gov/pmc/articles/PMC6581055/pdf/961.pdf

As more microbes are subjected to genome sequencing, and particularly with metagenome sequencing, it is becoming more important to separately identify plasmid DNA from chromosomal DNA.

## Database of plasmids

https://www.ebi.ac.uk/genomes/plasmid.html

While this page is not being updated, it still has a useful collection of microbial plasmid sequences.

